# Assessment of Quality of Life Among Patients Experiencing Pain: A Cross-Sectional Study at King Abdullah Medical City, Makkah, Saudi Arabia

**DOI:** 10.3390/ijerph22081191

**Published:** 2025-07-30

**Authors:** Maram Alshareef, Khadija AlJohari, Turki Alotaibi, Asim Alfahmi, Ahmad Bazarra, Somayah Malibary, Bayan Hashim Alsharif, Mokhtar Shatla

**Affiliations:** 1 Department of Community Medicine and Pilgrims Health Care, Faculty of Medicine, Umm Al-Qura University, Makkah 21955, Saudi Arabia; mmshatla@uqu.edu.sa; 2 University Clinics, Umm Al-Qua University, Makkah 21955, Saudi Arabia; ktjohari@uqu.edu.sa; 3 Faculty of Medicine, Umm Al-Qura University, Makkah 21955, Saudi Arabia; alotebiturky@gmail.com (T.A.); assimalfahmi7@gmail.com (A.A.); ahmadbazarrah@hotmail.com (A.B.); 4 Perioperative Medicine Administration, King Abdullah Medical City, Makkah 24331, Saudi Arabia; melibary.s@kamc.med.sa; 5 Hajj and Umrah Research and Epidemiology Administration, King Abdullah Medical City, Makkah 24331, Saudi Arabia; alsharif.b@kamc.med.sa

**Keywords:** chronic pain, quality of life, severity, physical, emotional, social

## Abstract

Chronic pain (CP) markedly impairs quality of life (QoL) and contributes to physical, psychological, and social dysfunction. In Saudi Arabia, limited research exists on CP and its impact on QoL. This cross-sectional questionnaire-based study conducted at King Abdullah Medical City, Makkah, over 3 months aimed to comparatively assess CP severity and the QoL between individuals who attended specialized pain clinics and those who did not. Data were collected from 250 participants by using a five-part questionnaire, including the RAND 36-Item Health Survey 1.0, to assess QoL. Statistical analysis included descriptive statistics and comparative analysis using the Statistical Package for the Social Sciences v22.0, with *p*-values of <0.05 considered significant. Most of the participants were female (56%), aged > 18 years (94.4%), Saudi nationals (88%), and married (72%). The most frequently reported pain site was the knee (33.6%). The mean scores for QoL domains were low, especially physical functioning (18.5), emotional well-being (38.4), and social functioning (38.8). Pain severity and poor general health were significantly associated with nationality, educational level, and clinic type. In conclusion, CP profoundly diminished QoL, particularly in terms of physical, emotional, and social aspects. Outcomes were influenced by factors such as educational level, employment, nationality, and clinical care settings.

## 1. Introduction

Approximately 20% of the global population is affected by chronic pain (CP), suggesting that approximately one in every five adults experiences CP. Moreover, the prevalence of high-impact CP is estimated to be approximately 8% [1]. In Saudi Arabia, limited research has been conducted regarding the prevalence of CP in the general population. However, a major study conducted in the middle region in Saudi Arabia revealed a CP prevalence of 46%, with approximately 4% of the individuals studied experiencing high-impact pain [2]. CP is characterized by persistent discomfort lasting anywhere from 3 to 6 months in any affected part of the body [3]. Affected individuals encounter numerous barriers that hinder effective CP management. Consequently, a substantial percentage of patients with CP remain dissatisfied with their treatment or do not receive any treatment at all [4,5]. Healthcare providers, unfortunately, commonly perceive CP as one of the most challenging and burdensome conditions to treat, primarily because of various obstacles such as logistical constraints within the healthcare system, limited treatment availability, challenges in accessing different management options, the prolonged duration of the condition, and the complexity involved in multidisciplinary care that can be provided for patients [6,7]. This perception affects patient satisfaction with CP management, leading to their complaints being misunderstood or dismissed, which further affects CP assessment and management. Consequently, patients with CP are often dissatisfied with the management of their condition or with their healthcare providers [8]. Inadequate management of CP has several consequences that can markedly impact an individual’s quality of life (QoL). This may lead to increased physical disability, as patients with CP may experience restricted movement owing to their condition and fear that movement could exacerbate pain [5]. This can result in muscle stiffness and affect other parts of the body, thereby increasing pain intensity, as described by the vicious cycle theory [9]. Furthermore, CP can trigger pain in other parts of the body by activating peripheral sensitization and exacerbating central sensitization [10]. Multiple studies have identified a significant association between chronic diseases, such as obesity, diabetes, and dyslipidemia, and movement restrictions, which can contribute to increased mortality among individuals with CP [11]. Moreover, studies have reported a significant increase in the incidence of coronary and cerebrovascular diseases in these individuals [12]. Regrettably, all the aforementioned consequences affect an individual’s psychological well-being, as a strong connection has been identified between pain and patients’ mental health, often resulting in depression and anxiety [13]. Furthermore, personal behaviors such as anger can isolate individuals from their families, particularly owing to the challenges others face in comprehending the suffering experienced by those with CP [14]. Numerous studies have investigated the QoL of individuals living with CP. A previous study revealed that CP markedly affects patients’ physical activity, daily work, sleep, personal relationships, and mood [15]. Additionally, there is an economic burden stemming from the resources used for pain management or a reduction in patient productivity, which, in turn, affects a country’s economy [16]. Several studies conducted in Saudi Arabia assessed the QoL of patients with specific conditions leading to CP, such as sickle cell disease and multiple sclerosis. These studies have consistently identified low QoL among the individuals affected by these conditions, indicating the need for more comprehensive management strategies [17,18]. However, no research has been undertaken in Saudi Arabia to evaluate the QoL of individuals with CP. Hence, this study was aimed at assessing the impact of the severity of CP on the QoL of individuals with CP and comparing those attending specialized pain clinics with those who did not.

## 2. Materials and Methods

### 2.1. Study Design and Setting

This cross-sectional study was conducted at King Abdullah Medical City (KAMC), Makkah, over a period of 3 months. The data collection took place from 1 June 2023 to 31 August 2023. Participants were purposively selected from the family medicine, orthopedic, physical therapy, and pain clinics. Recruitment was conducted directly from the waiting areas of these outpatient clinics at King Abdullah Medical City, Makkah, Saudi Arabia. The study was conducted in accordance with the Strengthening the Reporting of Observational Studies in Epidemiology (STROBE) guidelines for observational research.

### 2.2. Participants

Adults (aged ≥ 18 years) with a history of chronic pain (CP) who attended the aforementioned clinics at King Abdullah Medical City, Makkah, Saudi Arabia, were eligible to participate in the study. Written informed consent was obtained from all participants. Inclusion criteria required individuals to be 18 years or older, have experienced chronic pain for more than three months, and be able to understand and complete the study questionnaires independently or with minimal assistance. Exclusion criteria included patients with acute pain (lasting less than three months), those with cognitive impairment, psychiatric illness, or communication difficulties that interfered with questionnaire completion, as well as patients receiving palliative care.

### 2.3. Data Collection Tool

A structured five-section questionnaire was administered by trained volunteers to collect the following data:

- Demographics: age, sex, marital status, education, and occupation.
- Chronic Diseases: presence of comorbidities such as diabetes, hypertension, and cardiovascular diseases.
- Pain History: anatomical pain site(s) and frequency.
- QoL Assessment: the RAND 36-Item Health Survey 1.0 [19].
- Pain Management: treatment modalities, interventional therapies, and nonpharmacological therapies.

The RAND 36-Item Health Survey 1.0 assesses eight domains: physical functioning, role limitations due to physical and emotional health, energy/fatigue, emotional well-being, social functioning, pain, general health, and perceived health change. Scores range from 0 (worst) to 100 (best).

### 2.4. Data Analysis

The process of scoring the RAND 36-Item Health Survey involved two steps. Initially, precoded numeric values were recoded using a scoring key obtained from the Workplace Safety and Insurance Board. Each item was scored on a scale of 0 to 100, where 0 represented the lowest possible score and 100 signified the highest achievable score. These scores reflect the percentage of the total potential scores attained.

In the second step, items in the same scale were averaged, resulting in a total of seven scale scores. Any items left blank, because of missing data, were excluded from the computation of the scale scores.

Consequently, the scale scores represented the average for all items in the scale for which respondents provided answers. A calculated score exceeding 60% was considered to indicate a positive effect, whereas a mean score below 60% was deemed to indicate a negative effect.

SPSS v22.0 was used for statistical analysis. Categorical variables are presented as frequencies/percentages and continuous variables as mean ± standard deviation (SD) or median (IQR). Chi-square or Fisher’s exact tests were used to assess associations, with a *p*-value of <0.05 considered significant.

## 3. Results

### 3.1. Participant Characteristics

Overall, 250 participants were included in the study. The majority of the participants were female (56%, *n* = 140) and older than 18 years of age (94.4%, *n* = 236). Most participants were Saudi nationals (88%, *n* = 220) and married (72%, *n* = 180). More than half of the participants (57.6%, *n* = 144) had a high school or lower level of education. Regarding employment status, 64.2% (*n* = 160) of the participants were unemployed. Sixty-four percent of the participants reported having chronic diseases, with the most common conditions being diabetes (21.6%, *n* = 54), cardiovascular diseases (19.2%, *n* = 48), and hypertension (11.2%, *n* = 28). The participants’ demographic characteristics are shown in Table 1.

### 3.2. Pain Characteristics

The most reported pain sites were the knees (33.6%, *n* = 84), followed by the back (22.4%, *n* = 56) and feet (12.8%, *n* = 32). The distribution of chronic pain sites is shown in Table 2.

### 3.3. Quality of Life Assessment

The mean QoL scores across different domains were generally low, indicating reduced QoL among the participants. Low mean scores were observed for physical functioning (18.5) and emotional well-being (38.4). The scores for QoL and associated domains are listed in Table 3.

### 3.4. Factors Associated with QoL Domains

Table 4 shows the significant associations between sociodemographic factors and QoL domains:

- Educational level: Individuals with lower educational levels reported significantly worse pain (*p* = 0.001).
- Social status: Single participants had significantly higher pain severity (*p* = 0.022) and poorer general health (*p* = 0.029) than married or widowed participants.
- Nationality: Non-Saudi participants reported significantly worse physical functioning (*p* = 0.011) and higher pain severity (*p* = 0.036).
- Clinic type: Pain severity differed significantly (*p* = 0.011) across different clinical groups, with orthopedic clinic attendees reporting the highest levels.

Summary of Key Associations:

- Lower education = Worse pain
- Single marital status = Poorer general health
- Non-Saudi nationality = Worse physical functioning and pain
- Patients attending orthopedic clinics = Highest pain scores

**Table 4:** Association of sociodemographic factors with quality of life measures.

		Energy/Fatigue		*p* Value	Emotional Well-Being		*p* Value	Social Functioning		*p* Value	Pain		*p* Value	General Health		*p* Value
		Severe	Moderate		Severe	Moderate		Severe	Moderate		Severe	Moderate		Severe	Moderate	
Gender	Male	66 (60)	44 (40)	1	90 (81.8)	20 (18.2)	0.555	100 (90.9)	10 (9.1)	0.297	62 (56.4)	48 (43.6)	0.217	28 (25.5)	82 (74.5)	0.219
	Female	84 (60)	56 (40)		120 (85.7)	20 (14.3)		118 (84.3)	22 (15.7)		94 (67.1)	46 (32.9)		50 (35.7)	90 (644.3)	
Nationality	Saudi	130 (59.1)	90 (40.9)	0.57	190 (86.4)	30 (13.6)	0.07	188 (85.5)	32 (114.5)	0.214	144 (65.5)	76 (34.5)	0.05 *	7 0 (31.8)	150 (68.2)	0.775
	Non-Saudi	20 (66.7)	10 (33.3)		20 (66.7)	10 (33.3)		30 (100)	0		12 (40)	18 (60)		8 (26.7)	22 (73.3)	
Educational level	High school or less	68 (47.2)	76 (52.8)	0.002 ^&^	114 (79.2)	30 (20.8)	0.167	132 (91.7)	12 (8.3)	0.191	110 (76.4)	34 (23.6)	0.001 ^&^	54 (37.5)	90 (62.5)	0.251
	Bachelor’s/ Diploma	66 (75)	22 (25)		78 (88.6)	10 (11.4)		70 (79.5)	18 (20.5)		38 (43.2)	50 (56.8)		18 (20.5)	70 (79.5)	
	Master’s degree	6 (75)	2 (25)		8 (100)	0		8 (100)	0		2 (25)	6 (75)		2 (25)	6 (75)	
	PhD	10 (100)	0		10 (100)	0		8 (80)	2 (20)		6 (60)	4 (40)		4 (40)	6 (60)	
Working	Yes	56 (71.8)	2 2 (28.2)	0.07	72 (92.3)	6 (7.7)	0.064	70 (89.7)	8 (10.3)	0.428	42 (53.8)	136 (46.2)	0.358	16 (20.5)	62 (79.5)	0.098
	No	76 (54.3)	64 (45.7)		110 (78.6)	30 (21.4)		118 (84.3)	22 (15.7)		88 (62.9)	52 (37.1)		50 (35.7)	90 (64.3)	
Social status	Single	30 (78.9)	8 (21.1)	0.16	30 (85.6)	8 (21.1)	0.746	34 (89.5)	4 (10.5)	0.751	16 (42.1)	22 (57.9)	0.02 *	4 (10.5)	34 (89.5)	0.029 ^&^
	Married	104 (57.8)	76 (42.2)		154 (73.3)	26 (14.4)		158 (87.8)	22 (12.2)		112 (62.2)	68 (37.8)		58 (32.2)	122 (67.8)	
	Widower	16 (50)	16 (50)		26 (81,3)	6 (18.8)		26 (81.2)	6 (18.8)		28 (87.5)	4 (12.5)		16 (50)	16 (50)	
Clinic	FM	64 (62.7)	38 (37.3)	0.31	94 (92.2)	8 (7.8)	0.098	88 (86.3)	14 (13.7)	0.827	50 (49)	52 (51)	0.01 *	26 (25.5)	76 (74.5)	0.073
	Pain clinic	42 (67.7)	20 (32.3)		48 (77.4)	14 (22.6)		56 (90.3)	6 (9.7)		38 (61.3)	24 (38.7)		14 (22.6)	48 (77.4)	
	Orthopedic Clinic	22 (51.2)	42 (48.8)		68 (79.1)	18 (20.9)		74 (86)	12 (14)		68 (79.1)	18 (20.9)		3 8 (44.2)	48 (55.8)	

* Chi-square test; ^&^ Fisher exact test.

## 4. Discussion

In this study, we comparatively evaluated the impact of CP severity on the QoL between individuals who visited specialized pain clinics and those who did not. Our findings reaffirmed the substantial burden of chronic pain, with particularly severe impairments in physical functioning (mean: 18.5), role limitations due to physical health (mean: 0), emotional well-being (mean: 38.4), and social functioning (mean: 38.8). These scores fall far below normative RAND-36 population values, which typically exceed 70 for physical function and 80 for emotional well-being in healthy adults [19]. A ≥20-point reduction in any domain of the RAND-36 is considered clinically meaningful [20], suggesting that the deficits observed in this cohort represent profound impairments with real-world consequences, such as inability to work, limited social engagement, and compromised psychological health.

For example, the physical functioning score of 18.5 implies significant restrictions in basic mobility and self-care tasks. The absolute score of 0 in “role limitations due to physical health” reflects complete incapacity to perform occupational or daily responsibilities, underscoring the disabling nature of CP among our participants. This is especially relevant considering that 64.2% of the participants were unemployed, indicating not just physical impairment but also a potential loss of socioeconomic function. These findings are consistent with those of international and regional studies highlighting the broad and profound effects of CP on the daily activities and mental health of the affected individuals [21,22,23].

In our study sample, the most commonly affected body site was the knee (33.6%), followed by the back (22.4%) and feet (12.8%). This distribution aligns with that reported in a study by Fayaz et al. [24], who reported that musculoskeletal pain, particularly involving the knee and back, is among the most frequent complaints in individuals with CP. Similar findings were observed in a Saudi study by Almalki et al. [2], reinforcing the fact that lower limb and spinal pain dominate CP presentations in this region.

Interestingly, a notable finding of the current study was the significant association between educational level and pain severity; the participants with lower educational levels reported higher pain intensity. This finding is consistent with that reported by Wang et al. [25], who indicated that lower educational attainment was associated with higher rates of CP and worse health outcomes. The likely explanations include reduced access to health information, lower health literacy, and limited engagement with non-pharmacological management options among individuals with less education.

Another notable finding was the significant association between educational level and pain severity; the participants with lower educational levels reported higher pain intensity. This finding is consistent with that reported by Wang et al. [25], who indicated that lower educational attainment was associated with higher rates of CP and worse health outcomes. The likely explanations include reduced access to health information, lower health literacy, and limited engagement with non-pharmacological management options among individuals with less education.

Marital status critically affected CP, with single participants experiencing significantly higher pain severity and poorer general health than married or widowed participants. This finding supports the results of previous research which showed that lack of social support is crucial in exacerbating the psychological and physical consequences of CP [26]. Married individuals may benefit from spousal support, facilitating better coping strategies and adherence to treatment regimens; however, there is currently no supporting evidence in this regard [27].

Nationality also influenced outcomes, with non-Saudi participants reporting worse physical functioning and higher pain levels than Saudi participants. Similar disparities have been reported elsewhere, in which minority or non-national populations face greater barriers to healthcare access, leading to worse pain outcomes (predictive) [28]. In Saudi Arabia, variations were possibly influenced by the differences in insurance coverage, financial stability, social integration with the surroundings, work-related stress, and feelings of home sickness [28,29].

Interestingly, our data showed no statistically significant differences in emotional well-being or energy/fatigue between male and female patients, a finding that diverges from the previous literature, which often reports greater pain intensity, fatigue, and psychological distress among females [30,31]. Several explanations may account for this discrepancy. First, chronic pain may exert a ceiling effect on QoL—such that beyond a certain severity threshold, the burden becomes uniformly debilitating, regardless of sex. Second, sociocultural dynamics in Saudi Arabia, including gender norms around emotional expression and symptom disclosure, may influence how pain and emotional distress are reported. For example, men may underreport symptoms of fatigue or anxiety due to stigma, while women may minimize their complaints in clinical settings to avoid appearing weak or burdensome [32,33].

This cultural context complicates the interpretation of self-reported measures and highlights the need for more nuanced, culturally adapted assessment tools that can better capture distress in both genders. It also emphasizes the importance of clinician sensitivity to non-verbal cues and functional markers of suffering, especially in populations where psychological symptoms may be downplayed or somatized.

Pain severity and QoL outcomes varied meaningfully by clinical setting. Patients treated in orthopedic clinics reported the highest prevalence of severe pain (79.1%), followed by pain clinic patients (61.3%) and those attending family medicine clinics (49%). Although the differences between groups may appear modest, a relative 18% reduction in severe pain prevalence in pain clinic patients compared to orthopedic patients is clinically important. The literature suggests that even a 10–20% reduction in pain intensity correlates with improved sleep, mood, and functional independence, as well as reduced reliance on healthcare services [34,35]. These differences likely reflect the structural and therapeutic distinctions between the clinical settings. Specialized pain clinics typically adopt a multidisciplinary, biopsychosocial model that incorporates pharmacological interventional, physical, and psychological therapies. In contrast, orthopedic and family medicine clinics may focus on disease-specific management or symptom alleviation without holistic pain control [21,36]. Studies have consistently shown that multidisciplinary pain management is associated with better patient satisfaction, reduced pain scores, and improved QoL outcomes compared to standard care [37,38]. Nevertheless, even among those treated in pain clinics, QoL scores remained substantially below normative values. This suggests that while specialized care may reduce pain severity and somewhat improve function, it may not fully address the multidimensional impact of chronic pain. Chronic pain is a persistent, complex phenomenon that often resists resolution even under expert care, particularly when comorbid conditions such as depression or disability are present [3].

These findings collectively underscore the urgent need for broader integration of multidisciplinary pain services across general healthcare settings. The observed disparities between specialized and general clinics demonstrate that access to comprehensive pain management can yield tangible benefits in pain reduction and functional outcomes. Expanding access to these services, particularly in primary and orthopedic care, could help prevent the escalation of pain severity and improve long-term QoL for chronic pain patients.

Moreover, given the extreme deficits seen in key domains like physical function and role participation, there is a strong case for embedding rehabilitation services, mental health support, and vocational therapy within chronic pain care pathways. Policy efforts should also focus on training general practitioners in evidence-based pain management and enhancing referral systems to ensure timely access to specialized care.

## 5. Study Limitations

Despite offering valuable insights into the impact of chronic pain (CP) on quality of life (QoL) among patients in Saudi Arabia, several limitations must be acknowledged. The cross-sectional nature of this study precludes causal inferences between CP severity and QoL outcomes. It only captures associations at a single point in time, limiting the ability to determine whether poor QoL results from chronic pain or contributes to its severity. The single-center setting of the study may limit the generalizability of the findings. Participants were recruited purposively from outpatient clinics (family medicine, orthopedic, physical therapy, and pain clinics), which may introduce selection bias. Individuals already seeking medical care may differ systematically from those managing pain independently or not accessing healthcare services. The study is subject to sample demographics skew as the majority of the sample consisted of females (56%), unemployed individuals (64.2%), and Saudi nationals (88%), which may not reflect the diversity of CP sufferers in the general population and limits external validity. Data was collected via self-reported questionnaires, which are subject to recall bias and social desirability bias. The study did not adjust for potential confounding variables such as duration of pain, analgesic use, mental health diagnoses (e.g., depression or anxiety), or socioeconomic status, all of which could significantly influence QoL. A potential measurement bias is a possibility as a particularly unusual finding in this study was the zero-mean score in the RAND-36 domain of role limitations due to physical health. This could reflect true functional impairment or may point to a flaw in questionnaire interpretation, administration, or the cultural appropriateness of the tool.

## 6. Conclusions

This study assessed the quality of life among patients with chronic pain in Saudi Arabia and found that chronic pain significantly impairs multiple QoL domains—particularly physical, emotional, and social functioning. Factors such as educational level, employment status, nationality, and type of clinic attended were significantly associated with QoL outcomes. These findings highlight the need for tailored, multidisciplinary interventions to improve the well-being of individuals living with chronic pain.

## 7. Recommendations

- Healthcare Policy: Expansion of access to specialized pain management services, especially for disadvantaged groups.
- Clinical Practice: Implementation of multidisciplinary teams integrating physical, psychological, and social interventions.
- Future Research: Conducting large-scale, multicenter longitudinal studies to better understand the CP trajectory and management outcomes.
- Public Awareness: Improvement of patient and provider education regarding CP to reduce stigma and improve early intervention.

## Figures and Tables

**Table 1:** Demographic characteristics of the participants (*n* = 250).

Variable	Category	*n*	%
Sex	Male	110	44.0
	Female	140	56.0
Age	More than 18 years	236	94.4
	Less than 18 years	14	5.6
Nationality	Saudi	220	88.0
	Non-Saudi	30	12.0
Educational level	High school or less	144	57.6
	Bachelor’s degree/Diploma	88	35.2
	Master’s degree	8	3.2
	Doctorate	10	4.0
Marital status	Single	38	15.2
	Married	180	72.0
	Widowed	32	12.8
Occupational status	Employed	90	35.8
	Unemployed	160	64.2
Chronic Diseases	None	90	36.0
	Heart and vascular diseases	48	19.2
	Diabetes mellitus	54	21.6
	Hypertension	28	11.2
	Endocrine diseases	20	8.0
	Kidney disease	6	2.4
	Respiratory diseases	4	1.6

**Table 2:** Anatomical distribution of chronic pain (*n* = 250).

Pain Location	*n*	%
Knees	84	33.6
Shoulders	26	10.4
Back	56	22.4
Feet	32	12.8
Hips	10	4.0
Neck	14	5.6
Pelvis	8	3.2
Hands	8	3.2
Head	12	4.8

**Table 3:** Scores for quality of life and its domains based on the RAND 36-Item Health Survey 1.0.

Domain	Number of Items	Mean	SD	Minimum	Maximum
Physical functioning	9	18.5	15.7	0	50
Role limitations due to physical health	3	0	0	0	0
Energy/fatigue	4	50.4	20.3	0	100
Emotional well-being	5	38.4	16.9	0	90
Social functioning	2	38.8	18.4	0	100
Pain	2	46.6	24.6	0	100
General health	5	58.96	15.7	15	95

## Data Availability

The original contributions presented in this study are included in the article. Further inquiries can be directed to the corresponding author.

## Abbreviations

The following abbreviations are used in this manuscript:

CP	Chronic pain
QoL	Quality of life
KAMC	King Abdullah Medical City
SD	Standard deviation
IQR	Interquartile range
